# Safety and Efficacy of Highly Selective 5-Hydroxytryptamine Receptor 4 Agonists for Diabetic and Idiopathic Gastroparesis: A Systematic Review and Meta-Analysis of Randomized Controlled Trials

**DOI:** 10.7759/cureus.51851

**Published:** 2024-01-08

**Authors:** Parth Patel, Eli A Zaher, Himsikhar Khataniar, Mohamed A Ebrahim, Priyadarshini Loganathan

**Affiliations:** 1 Internal Medicine, Ascension Saint Joseph Hospital, Chicago, USA; 2 Medicine, St. John's Medical College, Bengaluru, IND; 3 Internal Medicine, University of Texas Health Science Center at San Antonio, San Antonio, USA

**Keywords:** #gastroparesis treatment, #prucalopride, #highly selective 5-ht4 agonist, #velusetrag, #felcisetrag

## Abstract

Gastroparesis significantly affects quality of life and healthcare expenditure. Effective treatment options are limited, and the utility of current prokinetic agents is inhibited by serious adverse effects. There exists an unmet need for prokinetic agents demonstrating both efficacy and an acceptable adverse effect profile. Highly selective 5-Hydroxytryptamine receptor 4 (5-HT4) agonists have exhibited clinical efficacy and safety in randomized controlled trials (RCTs). Consequently, we conducted a meta-analysis to comprehensively assess the safety and efficacy of these highly selective agents. Multiple databases, including PubMed, Scopus, and Embase, were systematically screened from inception until September 2023. Only RCTs evaluating the efficacy and safety of highly selective 5-HT4 agonists for gastroparesis were included. Key outcomes of interest included the pooled rates of Gastroparesis Cardinal Symptom Index (GCSI) scores, gastric emptying time (GET), and adverse event rates in each group. We adhered to standard meta-analysis methodology utilizing the random-effects model, with heterogeneity assessed by I^2^ statistics. Our analysis identified six RCTs, comprising 570 patients with diabetic (48%) or idiopathic (51%) gastroparesis, with mean ages of 46 and 45.9 years in the intervention and placebo groups, respectively. In the meta-analysis, highly selective 5-HT4 agonists demonstrated significantly superior pooled GCSI scores compared to placebo (mean difference: 4.283, (1.380, 7.186), p<0.05). Pooled GET was also significantly improved with 5-HT4 agonists compared to placebo (mean difference: 2.534, (1.695, 3.373), p<0.05). Although pooled rates of total adverse events were higher with 5-HT4 agonists (mean difference: 6.975, (1.042, 46.684), p<0.05), rates of specific adverse events such as diarrhea, abdominal pain, and headaches were comparable. In conclusion, this meta-analysis underscores a statistically significant improvement in GET and GCSI scores among patients receiving highly selective 5-HT4 agonists (Velusetrag, Felcisetrag, Prucalopride) for both diabetic and idiopathic gastroparesis. While the overall adverse effect profile is deemed acceptable, larger studies with extended follow-up periods are needed to investigate rare and/or serious adverse events. Moreover, future high-quality RCTs comparing the efficacy and safety of these novel agents with currently available agents are essential to further validate these findings.

## Introduction and background

Gastroparesis is a motility disorder characterized by symptoms and objective documentation of delayed gastric emptying of solid food in the absence of mechanical obstruction, which should be excluded by imaging studies or endoscopy. The chronic symptoms experienced by patients with gastroparesis may be associated with acute exacerbation of symptoms after the oral intake of food; these symptoms include postprandial fullness, nausea, vomiting, and upper abdominal pain [1]. The prevalence of gastroparesis in the United States is 267.7 per 100,000 adults. Global epidemiology is largely unknown, although population-based studies estimate that up to 1.8% of the population is affected [2]. Quality of life in patients with gastroparesis is impaired in comparison to the general population and is similar to that of patients with other chronic medical and psychological disorders. The degree of impairment in quality of life is related to the severity and duration of treatment [3].

Management of gastroparesis involves the correction or optimization of the underlying cause (such as diabetes, electrolyte, or nutritional deficiencies), dietary modifications, pharmacologic measures (prokinetics, antiemetics), and non-pharmacologic measures (gastric electrical stimulation, endoscopic or surgical interventions) [3]. Regardless of etiology, gastroparesis continues to burden healthcare utilization and expenditure, and it contributes significantly to the national healthcare bill [4]. Despite the considerable burden, metoclopramide is the only FDA-approved prokinetic agent available for gastroparesis in the USA, and evidence of its beneficial effects on quality of life is limited. The lack of newer options has led to the off-label use of macrolide antibiotics (e.g., erythromycin) and acetylcholinesterase inhibitors. Antiemetics such as ondansetron and promethazine are commonly used for symptom management [1].

There is an unmet need for more pharmacological options that are safer and more effective for gastroparesis patients. Several new pharmacological agents targeting specific receptors are currently being developed for gastroparesis, such as neurokinin 1 antagonist (tradipitant), synthetic ghrelin agonist (relamorelin), and highly selective 5-Hydroxytryptamine receptor 4 (5-HT4) agonists (velusetrag, prucalopride, felcisetrag) [5-12].

Here, we conducted a systematic review and meta-analysis of randomized controlled trials (RCTs) assessing the safety and efficacy of highly selective 5-HT4 agonists (velusetrag, prucalopride, felcisetrag) in patients with both diabetic and idiopathic gastroparesis [7-12].

## Review

Materials and methods

Study Design

A meta-analysis of RCTs was conducted to assess the safety and efficacy of highly selective 5-HT4 agonists (Velusetrag, Felcisetrag, Prucalopride) for diabetic and idiopathic gastroparesis. The Preferred Reporting Items for Systematic Reviews and Meta-Analyses (PRISMA) guidelines were followed to review the studied articles [13].

Study Selection

For this meta-analysis, we included studies on the safety and efficacy of highly selective 5-HT4 agonists, which include velusetrag, felcisetrag, or prucalopride for diabetic and/or idiopathic gastroparesis.

All the included studies are RCTs, including both full studies and conference abstracts. The exclusion criteria were as follows: (1) Single-patient case reports, case series, review articles, and editorials; (2) studies conducted in the pediatric (<18 years) population; (3) non-English language studies; (4) non-human/animal studies; (5) non-clinical laboratory studies; and (6) non-RCT studies.

PICO was determined as Population (P): patients with diabetic and/or idiopathic gastroparesis; Intervention (I): highly selective 5-HT4 agonist agents (velusetrag, felcisetrag, prucalopride); Comparison (C): we compared the efficacy and safety of highly selective 5-HT4 agonist agents (velusetrag, felcisetrag, prucalopride) to placebo for diabetic and/or idiopathic gastroparesis; and Outcomes (O): our primary outcome measures were improvement in gastric emptying time (GET), Gastroparesis Cardinal Symptom Index (GCSI), and adverse event rates.

Information Sources

On September 4, 2022, a systematic search was performed. We searched four databases: PubMed, Scopus, Embase, and Google Scholar. A snowball search was carried out by searching the reference lists of publications eligible for full-text review and using Google Scholar to locate and screen studies citing them.

Search Strategy

The literature was searched for the concepts of gastroparesis, 5-HT4 agonists, highly selective 5-HT4 agonists, velusetrag, felcisetrag, prucalopride, and RCTs. The search was conducted using generic free-text search terms developed based on the Patient/Population, Intervention, Comparison group/Control, and Outcomes (PICO) model to define the clinical question and aid in finding clinically relevant evidence in the literature. 

For Population (P), the terms used were "GASTROPARESES" OR "GASTROPARESIS" OR "GASTRIC STASIS" OR "GASTRIC STASES". For Intervention (I), the terms were "SEROTONIN 5 HT4 RECEPTOR AGONISTS" OR "5-HT4 AGONISTS" OR "5 HT4 AGONISTS" OR "AGONISTS, 5-HT4" OR "5-HT4 AGONIST" OR "5 HT4 AGONIST" OR "PRUCALOPRIDE" OR "MOTEGRITY" OR "RESOTRAN" OR "RESOLOR" OR "VELUSETRAG" OR "FELCISETRAG".

The search terms were kept broad to encompass all possibilities for applicable studies. All studies published from inception to September 4, 2023, were retrieved to assess their eligibility for inclusion in this study. We limited our search to full-text articles in the English language. To find additional eligible studies, reference lists of included citations were cross-checked.

Selection Process

**Figure 1:**
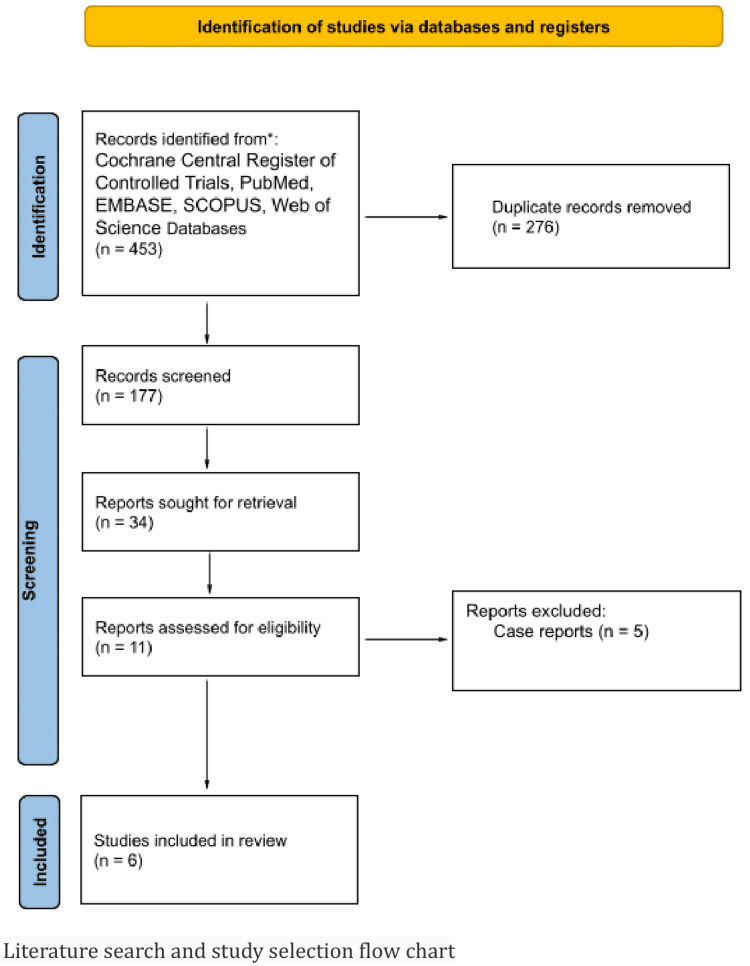
PRISMA flow diagram of the inclusion criteria of studies found eligible in the meta-analysis. PRISMA: Preferred Reporting Items for Systematic Reviews and Meta-Analyses.

All records identified by our search strategy were exported to EndNote. Duplicate articles were removed from the list. The title and abstract of studies from the primary search were independently screened by two authors (PP and EAZ). Based on predetermined inclusion and exclusion criteria, studies that did not address our specific research question were excluded. The full texts of the initially screened-in articles were then reviewed for relevant information. Any discrepancy in article selection was resolved by mutual consensus. Additional relevant articles were manually searched from the bibliographic sections of the selected articles, as well as from systematic and narrative reviews on the topic. The search methodology was documented in the PRISMA flow chart, which depicted the studies that were included as well as those excluded with accompanying justifications, as illustrated in Figure 1.

Data Abstraction and Quality Assessment

Two authors (EAZ and HK) independently abstracted data from the studies using a standardized form. Andrews et al. and Carbone et al. are crossover trials; we incorporated data from both intervention periods as such trials were a parallel group of intervention vs. placebo as per Cochrane guidelines [10-11,14]. Quality assessment was done independently by two authors (MAE, EAZ) using the Jadad scale for cohort studies to assess the quality of the studies [15]. The details are summarized in Table 1.

**Table 1:** Jadad scale for all included cohort studies to assess the quality of studies.

Study	Randomization			Blinding			Account of all patients	Score	Quality
	Randomization mentioned: +1	Randomization appropriate: +1	Inappropriate method of randomization: –1	Blinding mentioned: +1	Method appropriate: +1	Method inappropriate: –1	All points accounted for: +1		
Abell, 2021 [7]	1	1	0	1	1	0	1	5	High
Kuo, 2021 [8]	1	1	0	1	1	0	1	5	High
Ahn, 2015 [9]	1	1	0	0	0	0	1	3	Medium
Carbone, 2019 [10]	1	1	0	1	1	0	1	5	High
Andrews, 2021 [11]	1	1	0	1	1	0	1	5	High
Chedid, 2021 [12]	1	1	0	1	1	0	1	5	High

Outcomes Assessed

The outcomes measured were the reduction in GET, reduction in the GCSI, and adverse event rates in patients with diabetic or idiopathic gastroparesis receiving highly selective 5-HT4 agonists (Velusetrag, Felcisetrag, Prucalopride) in comparison to placebo.

Statistical Analysis

Following the methods suggested by DerSimonian and Laird, pooled efficacy rates with the corresponding 95% confidence interval (CI) were calculated by logit-transformation using a random-effects model. Heterogeneity between study-specific estimates was assessed using the Cochrane Q statistical test for heterogeneity and the I^2^ statistics [16]. The publication bias assessment was deferred as the number of studies analyzed was less than ten. All analyses were performed using Comprehensive Meta-Analysis (CMA) software, version 4 (BioStat, Englewood, New Jersey).

Results

The initial search yielded 453 references. After the removal of duplicates, a total of 177 studies, including full articles and abstracts, underwent title and abstract screening. Of the 11 studies initially felt to be appropriate, five were case reports, which were excluded from the final analysis. A total of six studies based on inclusion and exclusion criteria were included. All six studies are RCTs. One out of the six included studies is a conference abstract - the study selection flow chart is illustrated in Figure 1.

Six studies met the inclusion criteria, comprising 570 patients (400 in the intervention group and 170 in the placebo group). The mean ages of patients were 46 and 45.9 years in the intervention and placebo groups, respectively. Etiologies of gastroparesis reported by five studies (a total of 468 patients) were diabetic (48% of patients) or idiopathic (51% of patients). Kuo et al. and Abell et al. studied Velusetrag 5 mg, 10 mg, and 30 mg once daily oral doses for the duration of one and three weeks respectively, while Ahn et al. studied Velusetrag 5 mg, 10 mg, and 15 mg for the duration of one week [7-9]. Carbone et al. and Andrew et al. studied Prucalopride 2 mg and 4 mg oral daily respectively for a duration of four weeks [10-11]. Chedid et al. studied Felcisetrag 0.1 mg, 0.3 mg, and 1 mg IV infusion daily for three days [12]. Study characteristics are summarized in Table 2.

**Table 2:** Summary of all RCTs included in meta-analysis. RCTs: randomized controlled trials.

Study ID	Study Information	Drug; dose	Gastroparesis etiology	Total study population (n)	Intervention (n)	Placebo (n)	Treatment duration
Abell, 2021 [7]	Randomized, placebo-controlled, double-blinded	Velusetrag; 5, 15, 30 mg	Diabetic, idiopathic	232	173 (59, 56, 58)	59	4 weeks
Kuo, 2021 [8]	Randomized, placebo-controlled, double-blinded	Velusetrag; 5, 15, 30 mg	Diabetic, idiopathic	102	76 (26, 25, 25)	26	1 week
Ahn, 2015 [9]	Randomized, placebo-controlled	Velusetrag; 5, 15, 30 mg	Diabetic, idiopathic	102	76 (26, 25, 25)	26	1 week
Carbone, 2019 [10]	Randomized, placebo-controlled, double-blinded, cross-over	Prucalopride; 2 mg	Diabetic, idiopathic	68	34	34	4 weeks
Andrews, 2021 [11]	Randomized, placebo-controlled, double-blinded, cross-over	Prucalopride, 4 mg	Diabetic, connective tissue disease	30	15	15	4 weeks
Chedid, 2021 [12]	Randomized, placebo-controlled, double-blinded	Felcisetrag; 0.1 mg, 0.3 mg, 1.0 mg	Diabetic, idiopathic	36	26 (10, 9, 7)	10	3 days

Pooled outcomes

Gastroparesis Cardinal Symptom Index (GCSI)

**Figure 2:**
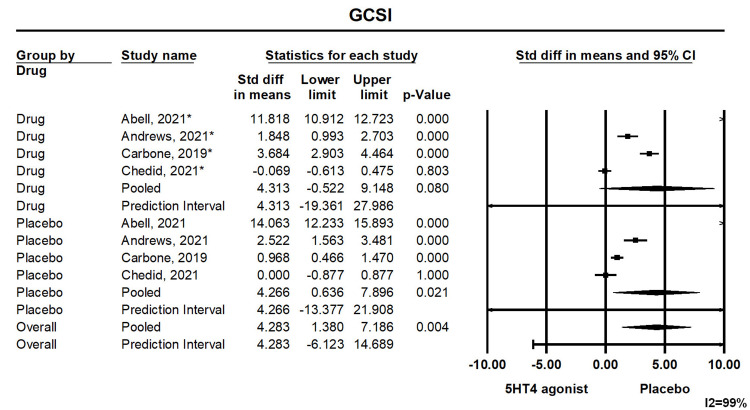
Forest Plot 1. Statistically significant improvement in GCSI with 5-HT4 agonists compared to placebo. Heterogeneity I^2^=99%. GCSI: gastroparesis cardinal symptom index; 5-HT4: 5-hydroxytryptamine receptor 4; CI: confidence interval.

Patients receiving highly selective 5-HT4 agonists demonstrated significant improvement in the GCSI as compared to placebo (mean difference: 4.283, (1.380, 7.186), p<0.05). Forest plot 1 is illustrated in Figure 2.

Gastric Emptying Time (GET)

**Figure 3:**
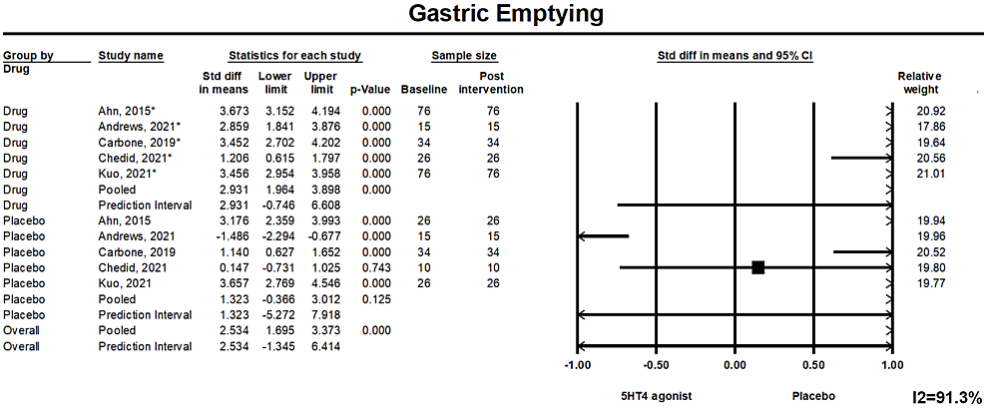
Forest Plot 2. Statistically significant improvement in GET with 5-HT4 agonists compared to placebo. Heterogeneity I^2^=91.3%. GET: gastric emptying time; 5-HT4: 5-hydroxytryptamine receptor 4; CI: confidence interval.

Pooled GET in patients receiving highly selective 5-HT4 agonists was significantly less in comparison to the placebo group (mean difference: 2.534, (1.695, 3.373), p<0.01). Forest plot 2 is illustrated in Figure 3.

Adverse Event

**Figure 4:**
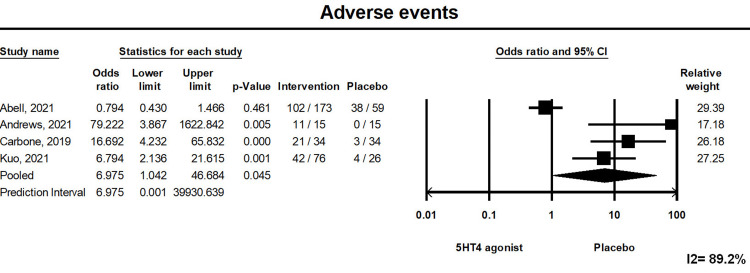
Forest Plot 3. The pooled risk of developing adverse events was greater in patients receiving 5-HT4 agonists compared to placebo. Heterogeneity I^2 ^= 89.2%. 5-HT4: 5-hydroxytryptamine receptor 4; CI: confidence interval.

**Figure 5:**
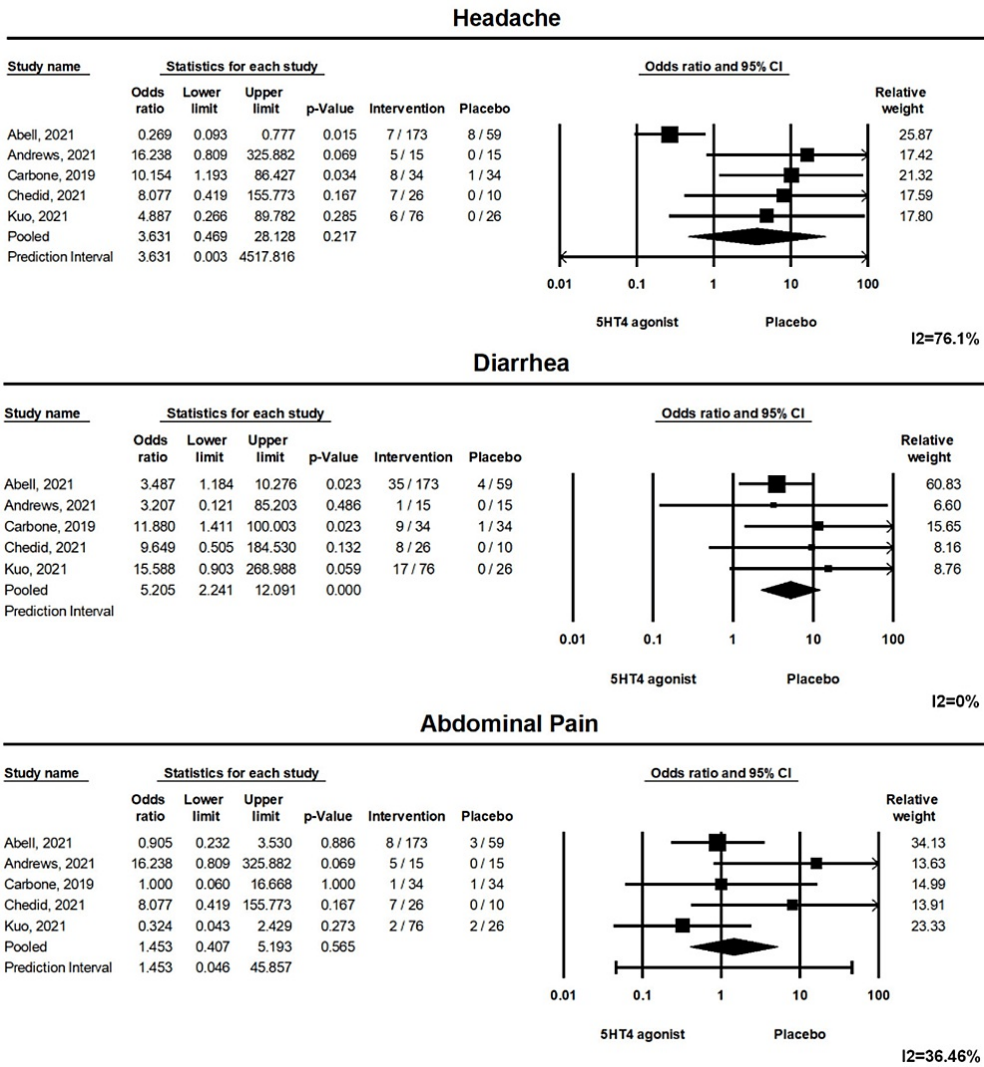
Forest plot showing pooled risk of specific adverse events headaches, diarrhea, and abdominal pain are comparable between the groups. 5-HT4: 5-Hydroxytryptamine receptor 4; CI: Confidence Interval

The pooled risk of developing adverse events was greater in patients receiving 5-HT4 agonists (pooled risk ratio: 6.975, (1.042, 46.684), p<0.05). Forest plot 3 is illustrated in Figure 4. However, the pooled risk of specific adverse events such as diarrhea, abdominal pain, and headaches was comparable between the groups (Figure 5). A total of 12 patients from the intervention group had serious side effects, out of which only one patient (who developed volvulus on day 18 of prucalopride) had serious side effects related to the intervention itself [10]. Out of these three highly selective 5-HT4 agonists, felcisetrag and velusetrag affect the QTc interval. Chedid et al. (Felcisetrag) and Abell et al. (Velusetrag) reported an effect on the QTc interval; none of the patients had a QTc >500 milliseconds throughout the study period [7,12].

The pooled outcomes of the meta-analysis are summarized in Table 3. 

**Table 3:** Summary of pooled outcomes from meta-analysis. 5-HT4: 5-hydroxytryptamine receptor 4.

Outcomes	Highly selective 5-HT4 agonist to placebo	I^2^%	p-Value	Studies (n)
Gastric emptying time	Pooled MD: 2.534 (1.695, 3.373)	91.30%	<0.001	5
Gastroparesis cardinal symptom index	Pooled MD: 4.283 (1.380, 7.186)	99%	0.004	6
Adverse events (all)	Pooled RR (95%CI): 6.975 (1.042, 46.684)	89.2%	0.045	4
Diarrhea	Pooled RR (95%CI): 5.205 (2.241, 12.091)	0%	<0.001	5
Abdominal pain	Pooled RR (95%CI): 1.453 (0.407, 5.193)	36.50%	0.565	5
Headache	Pooled RR (95%CI): 3.631 (0.469, 28.13)	76.1%	0.217	5

Validation of meta-analysis results

Sensitivity Analysis

To assess the possible dominant effect of individual studies on the meta-analysis, we excluded one study at a time and analyzed its effect on the main summary estimate. We did not find any single study significantly affecting the outcome or the heterogeneity.

Heterogeneity

We assessed the dispersion of the calculated rates using the I^2^ percentage values. Based on the I^2^ analysis for heterogeneity, substantial heterogeneity was noted for the improvement in the GCSI score, improvement in GET, and adverse events rate. The I2 values for the pooled rates are summarized in Table 3.

Prediction Interval

We assessed the dispersion of the calculated rates using the prediction interval (PI) and I^2^ percentage values. The PI gives an idea of the range of the dispersion, and I^2^ tells us what proportion of the distribution is true versus chance. The prediction interval and I2 are provided in forest plots (Figures 2-4).

Publication Bias

A publication bias assessment was deferred as the total number of studies included in the final analysis was less than 10 [17].

Discussion 

In this systematic review and meta-analysis, we demonstrated the safety and efficacy of highly selective 5-HT4 agonists compared with placebo for several patient-important outcomes in the treatment of diabetic and/or idiopathic gastroparesis: GCSI, GET, and adverse effects. Overall analyses showed significant benefit from treatment with highly selective 5-HT4 agonists when compared with placebo in terms of improvement in the GCSI and GET. Six RCTs fulfilling inclusion criteria were identified. Pooled data demonstrated significant improvement in GET and the GCSI with an acceptable side effect profile favoring highly selective 5-HT4 agonist agents.

Andrews et al. failed to show symptomatic benefits from prucalopride 4 mg in patients with diabetic gastroparesis despite significant enhancement in GET [11]. Carbone et al. reported significant symptomatic benefits from prucalopride 2 mg in the entire prucalopride group and further in sub-analyses of the idiopathic gastroparesis patient population [10]. Both Kuo et al. and Abell et al. reported enhanced GET with velusetrag, with disproportionate improvement in symptoms [7-8]. Chedid et al. observed a dose-dependent increase in GET with felcisetrag [12]. In our analysis, the mean difference of 2.5 minutes (1.695, 3.373) p<0.01 in GET with 5-HT4 agonists in comparison to placebo is statistically significant but notably small. Meta-analysis by Vijayvargiya and colleagues showed that an acceleration of GET >20.4 minutes is associated with clinically meaningful symptom improvement [18]. Clinical improvement as demonstrated by improvement in the GCSI score when compared to placebo (mean difference: 4.283, [1.380, 7.186], p<0.05) in our analysis is substantial and statistically significant.

The disparity between the dose-response relationship for GE acceleration and symptomatic improvement is not unexpected and has previously been observed in meta-regression analysis of 34 placebo-controlled studies of prokinetics agents by Janssen and colleagues, which did not show a correlation between reduction in GET and symptom improvement [19]. The failure to improve clinical symptoms at higher doses is explained by the fact that prokinetic agents decrease fundic accommodation of food and drive it too quickly, which provokes symptoms [11,18]. Additionally, the failure to improve symptoms despite GE acceleration in some patients can be attributed to the gastrointestinal side effects produced by the prokinetic agents, which are perceived as a lack of symptom improvement. The relationship between gastric emptying rate and symptoms is complex, and it is important to note that acceleration of GET will not always lead to symptomatic improvement [20].

All three highly selective 5-HT4 agonists included in the meta-analysis are generally well tolerated in gastroparesis patients. Most adverse effects from all three agents are gastrointestinal tract-related, such as diarrhea and abdominal cramps due to enhanced gastrokinetic effects produced by these agents. None of the patients who received velusetrag developed serious side effects directly related to the drug [7-9]. Kuo et al. observed a paradoxical trend where patients advancing to higher doses of velusetrag experienced fewer GI-related adverse events, including diarrhea, despite a more significant reduction in gastric emptying (GE) time [8]. This suggests potential desensitization to side effects while still benefiting from improved GE. In contrast, Abell et al. reported a dose-dependent increase in the frequency of GI-related side effects with velusetrag, possibly due to off-target effects from higher doses inducing colonic contractions [7,20]. Except for one patient who developed volvulus, all other participants who received prucalopride reported minor and transient drug-related adverse effects such as headache, diarrhea, and abdominal cramps [10,11]. Felcisetrag was also well tolerated across all three doses (0.1 mg, 0.3 mg, and 1 mg) without serious adverse events directly related to the drug [12]. Cardiovascular side effects, especially QTc prolongation, are a problem with selective 5-HT4 agonists. Abell et al. and Chedid et al. reported incidents of QTc prolongation following the use of velusetrag and felcisetrag respectively. Few patients developed prolonged QTc, but none of the patients had QTc exceeding 500 milliseconds [7,12].

These highly selective 5-HT4 agonists are new and there is a dearth of literature assessing their efficacy for gastroparesis and adverse effect profile. There is no prior meta-analysis assessing the safety and efficacy of highly selective 5-HT4 agonists for gastroparesis. All included studies are RCTs and of high quality as per the Jadad scale [15]. Our study has several limitations. Firstly, the studies incorporated into our analysis have examined these highly selective 5-HT4 agonist agents over durations ranging from three days to four weeks. This limited timeframe restricts the assessment of potential long-term adverse effects associated with the use of these agents. Secondly, three out of the six studies included in our analysis are Phase 2 studies and have not explored symptom improvement, which stands as one of the most crucial study outcomes for determining applicability. The absence of data on symptom improvement from these Phase 2 studies diminishes the depth of our analysis in evaluating the overall clinical impact of the interventions under investigation. Thirdly, given all included studies are the earliest RCTs for respective drugs, they are prone to the Proteus phenomenon, contributing to heterogeneity.

## Conclusions

In conclusion, this systematic review and meta-analysis supports a role for highly selective 5-HT4 agonists in the treatment of gastroparesis, given the finding of beneficial effects across multiple patient-important outcomes with a relatively favorable safety profile. Although this evidence is derived from RCTs, it is considered to be of moderate quality due to heterogeneity and the fact that novel therapies usually have an exaggerated effect size in their earlier publications, known as the Proteus phenomenon. Continued development of this class of drugs and ongoing investigation into the long-term safety and efficacy of these agents are needed.

## References

[1] Camilleri M, Kuo B, Nguyen L (2022). ACG clinical guideline: gastroparesis. Am J Gastroenterol.

[2] Ye Y, Yin Y, Huh SY, Almansa C, Bennett D, Camilleri M (2022). Epidemiology, etiology, and treatment of gastroparesis: real-world evidence from a large US national claims database. Gastroenterology.

[3] Camilleri M, Chedid V, Ford AC (2018). Gastroparesis. Nat Rev Dis Primers.

[4] Wadhwa V, Mehta D, Jobanputra Y, Lopez R, Thota PN, Sanaka MR (2017). Healthcare utilization and costs associated with gastroparesis. World J Gastroenterol.

[5] Carlin JL, Lieberman VR, Dahal A (2021). Efficacy and safety of tradipitant in patients with diabetic and idiopathic gastroparesis in a randomized, placebo-controlled trial. Gastroenterology.

[6] Camilleri M, Acosta A (2015). Emerging treatments in neurogastroenterology: relamorelin: a novel gastrocolokinetic synthetic ghrelin agonist. Neurogastroenterol Motil.

[7] Abell TL, Kuo B, Esfandyari T (2023). A randomized, double-blind, placebo-controlled, phase 2b study of the efficacy and safety of velusetrag in subjects with diabetic or idiopathic gastroparesis. Neurogastroenterol Motil.

[8] Kuo B, Barnes CN, Nguyen DD (2021). Velusetrag accelerates gastric emptying in subjects with gastroparesis: a multicentre, double-blind, randomised, placebo-controlled, phase 2 study. Aliment Pharmacol Ther.

[9] Ahn A, Barnes C, Shaywitz D, Grimaldi M, Canafax DM (2015). Su1426 velusetrag improves gastric emptying time in subjects with diabetic or idiopathic gastroparesis. Gastroenterology.

[10] Carbone F, Van den Houte K, Clevers E (2019). Prucalopride in gastroparesis: a randomized placebo-controlled crossover study. Am J Gastroenterol.

[11] Andrews CN, Woo M, Buresi M (2021). Prucalopride in diabetic and connective tissue disease-related gastroparesis: randomized placebo-controlled crossover pilot trial. Neurogastroenterol Motil.

[12] Chedid V, Brandler J, Arndt K (2021). Randomised study: effects of the 5-HT(4) receptor agonist felcisetrag vs placebo on gut transit in patients with gastroparesis. Aliment Pharmacol Ther.

[13] Page MJ, McKenzie JE, Bossuyt PM (2021). The PRISMA 2020 statement: an updated guideline for reporting systematic reviews. Syst Rev.

[14] Higgins JPT, Eldridge S, Li T (2023). Including variants on randomized trials. Cochrane Handbook for Systematic Reviews of Interventions, Version 6.4.

[15] Jadad AR, Moore RA, Carroll D, Jenkinson C, Reynolds DJ, Gavaghan DJ, McQuay HJ (1996). Assessing the quality of reports of randomized clinical trials: is blinding necessary?. Control Clin Trials.

[16] DerSimonian R, Laird N (1986). Meta-analysis in clinical trials. Control Clin Trials.

[17] Page MJ, Higgins JPT, Sterne JAC (2023). Assessing risk of bias due to missing results in a synthesis. Cochrane Handbook for Systematic Reviews of Interventions, Version 6.4.

[18] Vijayvargiya P, Camilleri M, Chedid V, Mandawat A, Erwin PJ, Murad MH (2019). Effects of promotility agents on gastric emptying and symptoms: a systematic review and meta-analysis. Gastroenterology.

[19] Janssen P, Harris MS, Jones M (2013). The relation between symptom improvement and gastric emptying in the treatment of diabetic and idiopathic gastroparesis. Am J Gastroenterol.

[20] Tack J, Goelen N, Carbone F (2020). Prokinetic effects and symptom relief in the pharmacotherapy of gastroparesis. Gastroenterology.

